# Parent–adolescent discussion on sexual and reproductive health issues and its associated factors among parents in Sawla town, Gofa zone, Ethiopia

**DOI:** 10.1186/s12978-022-01414-w

**Published:** 2022-05-02

**Authors:** Nigatu Teferi Malango, Tadele Yohannes Hegena, Netsanet Abera Assefa

**Affiliations:** 1 Gofa Zone, Sawla Town Health Office, Sawla, Ethiopia; 2 Department of Public Health, College of Medicine and Health Sciences, Wachemo University, Hossana, Ethiopia; 3 School of Public Health, College of Medicine and Health Sciences, Hawassa University, Hawassa, Ethiopia

**Keywords:** Parent–adolescent discussion, Sexual and reproductive health, Southern Ethiopia

## Abstract

**Background:**

Parent–adolescent discussion on sexual and reproductive health (SRH) issues leads to increased awareness on reproductive health matters and reduces risky behaviors among adolescents and also contributes to negative SRH outcomes. The aim of this study was to assess parent–adolescent discussion on SRH issues and its associated factors in Sawla town, Southern Ethiopia.

**Methods:**

A community-based cross-sectional study was conducted among parents of 10–19-year-olds in Sawla town from March to April 2020. A simple random sampling technique was employed to select 697 study subjects. Data were collected using a pretested structured questionnaire, adapted from the 2016 Ethiopia Demographic Health Survey and other published literature. The questionnaire consisted of questions about socio-demographic factors, knowledge about SRH issues, attitudes towards SRH issues, and discussions between parents and adolescents concerning SRH issues and was administered by face-to-face interviews p-value < 0.05 and 95%CI for adjusted odds ratios (AOR) were used to declare the significance of the associations.

**Results:**

Of the total participants, 168 (25.7%) respondents had reported discussion about at least two components of SRH issues in the last 6 months prior to the study. Multiple logistic regression model revealed that attending primary education (AOR 3, 95% CI: 1.10, 7.70), secondary education (AOR 9, 95% CI: 3.87, 20.64) and diploma and above (AOR 22.33, 95% CI: 9.49, 32.56), monthly income of above 2000 Ethiopian Birr (AOR 2.40, 95% CI: 1.30, 4.55), good knowledge (AOR 2, 95% CI: 1.14, 3.50)and positive attitude (AOR 4, 95% CI: 1.57, 10) towards SRH issues were statically significant factors associated with increased parent–adolescent discussion about SRH.

**Conclusion:**

The occurrence of parent–adolescent SRH discussion is low in Sawla town. Educational status, average monthly income, knowledge and attitude on SRH issues were major factors affecting parent–adolescent discussion on SRH issues. Continuous capacity building on SRH issues for parents to increase the level of parental knowledge and attitude about SRH issues and sustainable advocacy should be provided about the importance of parent to the adolescent discussion. Moreover, it is important to support and scale up the adult learning program to decrease the level of illiteracy.

## Plain English summary

The health threats for adolescents today are predominantly behavioral rather than biomedical and more of today’s adolescents are involved in health behavior with the potential for serious consequences. In most cultures, parents and family members are an influential source of knowledge, beliefs, attitudes, and values for children and youth. Open parent–adolescent discussion on sexual and reproductive health (SRH) issues often leads to increased awareness on reproductive health matters and reduces risky behaviors among adolescents and also is the base to reduce bad reproductive health outcomes. This study provides an overview of parent–adolescent discussion on SRH issues among parents in Sawla town in Southern Ethiopia. We found that only about one-fourth (25.7%) of the parents discuss at least two components of SRH issues with their children in the last 6 months prior to the study. Educational status, average monthly income, knowledge, and attitude on SRH issues were major factors for these disparities. Knowing the level of parent–adolescent discussion on SRH issues with their children and factors affecting the discussion may help concerned bodies to design Continuous capacity building and life skill training programs on SRH issues for parents and adolescents.

## Background

Adolescence is a continuum of physical, cognitive, behavioral, and psychosocial change that is characterized by increasing levels of individual autonomy, a growing sense of identity and self-esteem, and progressive independence from adults [1]. According to World Health Organization (WHO), it is one of the most rapid and formative phases of human development. These distinctive physical, cognitive, social, emotional, and sexual development demands special attention in national development policies and programs [2].

Reproductive health implies that people are able to have a satisfying and safe sex life and that they have the ability to reproduce and the freedom to decide if, when, and how often to do so [3]. SRH regardless of age and sex concerns everyone for a large part of their lives. This issue starts to affect people’s lives in adolescenc when they are familiar that they are starting to change from being children to adulthood [4].

Parent–adolescent discussion on SRH issues remains a challenging subject of discussion in many traditional communities as the social environment in them still constrains the exchange of information on such issues [5].

The results of adolescent sexual behavior are a huge burden both for the adolescent and society. The problem is not that adolescents are sexually active but rather that they have little preparation and guidance in developing responsible sexual behavior. Developmentally, adolescents reach physical maturity before they are cognitively able to appreciate the consequences of their behavior [6].

An adolescent’s primary source of information regarding sexuality is his or her peer group, all of whom are experiencing and reinforcing the same behaviors. The family, the major fraternize of other behaviors, is not as powerful a force in shaping responsible sexual behavior because of parental discomfort with sex education and sexual discussions. Parents most often respond with their hopes that their children will grow into adulthood without unintended pregnancy or a sexually transmitted infection (STI). They sometimes follow this with the hope that their kids will never be a victim or perpetrator of intimate partner violence or other sexual abuse [7]. This is the result of a social environment in which sex is frequently portrayed but rarely linked with responsible behavior or accurate, non-judgmental information [6].The magnitude of parent–adolescent discussion about SRH issues is variable from country to country, being highest in United States of America (70.6%), Mexico (83.1%) and very low in countries like Myanmar (6.8%) and India (13%) in South and East Asia [8–11]. A study conducted in Nigeria shows that only 12.3% of parents discuss sexual matters with their adolescent girls [12], while another study in Alamata, Ethiopia shows that discussion was 14.7% with mothers, 3.9% with fathers, 15% with brothers or sisters, 23.4% with the same sex, 5.7% with the opposite sex and 15% discussed with health personnel [13].

Adolescents are exposed to various sexual and reproductive risks such as early sexual debut, unprotected sex, early pregnancy, school dropout and STI/HIV due to lack of communication with their parents. Worldwide, HIV/ is the leading causes of disease burden for young people (those aged 10–24 years) [14]. Half the newly acquired HIV infections occur in young people, with most of those affected living in developing countries [1, 15]. Different studies showed that parents lack of interest to discuss, feeling ashamed, culturally not acceptable, lack of communication skills, lack of awareness and belief that discussion will initiate sex as factors affecting parent–adolescent discussion on SRH matters [16–18]. Poor parental involvement in preparing adolescent people for SRH also put adolescents’ victim of unwanted problems [19].

The government of Ethiopia developed Adolescent and Youth Reproductive Health (AYRH) Strategy in 2006, implemented from 2007 to 2015 and revised from 2016 to 2020. The strategy encouraged adolescents and youth to utilize available health services. The strategy mention the importance of parent–adolescent communication on SRH issues, but the government has not made an effort to encourage or facilitate it [19].

Even though discussion on SRH issues is a public health concern among adolescents, it has not received due attention in Ethiopia. The Parent–adolescent discussion and associated factors were not well studied in Ethiopia especially in Southern part. The previous studies done were school based and focused discussion on SRH issues only from adolescents’ point of view. Since this study was community based and from parents’ point of view, it will play major role in filling research gaps on this issue. Therefore, this study aimed to investigate the extent of parent–adolescent discussions on SRH and the parental factors that affect the likelihood of these discussions in Sawla Town, Southern Ethiopia.

## Materials and methods

### Study area

The study was conducted in Sawla Town, located in Southern nations and nationalities and People Regional State (SNNPR), Ethiopia. It is 310 km far from Hawassa, capital city of South Regional State in Ethiopia and 549 km away from Addis Ababa, the capital city of Ethiopia. Sawla has total population of 46,957, of which 51% are females. According to Ethiopian Health and Demographic Survey (EDHS) 2019, around 25.4% (11,927) adolescents were expected to live in the town. The town has 10 kebeles (smallest administrative unit) with 9582 households; of these 3903 households have adolescents. Sawla has one General Hospital, one public health center, two government clinics, nine private primary clinics, two private medium clinics, two pharmacies and seven drug stores which provide maternal and other health services to the population. Only one health center provides the adolescent and youth friendly services [20].

### Study design and period

A community based cross-sectional study was conducted from March to April, 2020.

### Study participants

The source population was all parents living in Sawla town who had children between 10 and 19 years (adolescent) of age. Parents who were unable to communicate or severely ill at the time of data collection were excluded from the study.

### Sample size determination and sampling procedure

Sample size was calculated using double population proportion estimation formula in EPI-INFO version 7 based on key variables taken from previous studies. The assumptions are 95% confidence interval, 80% power of test and ratio of exposed to unexposed = 1:1. The calculated sample size was 634. After adding a non-response rate of 10%, the final sample size became 697.

**Fig. 1:**
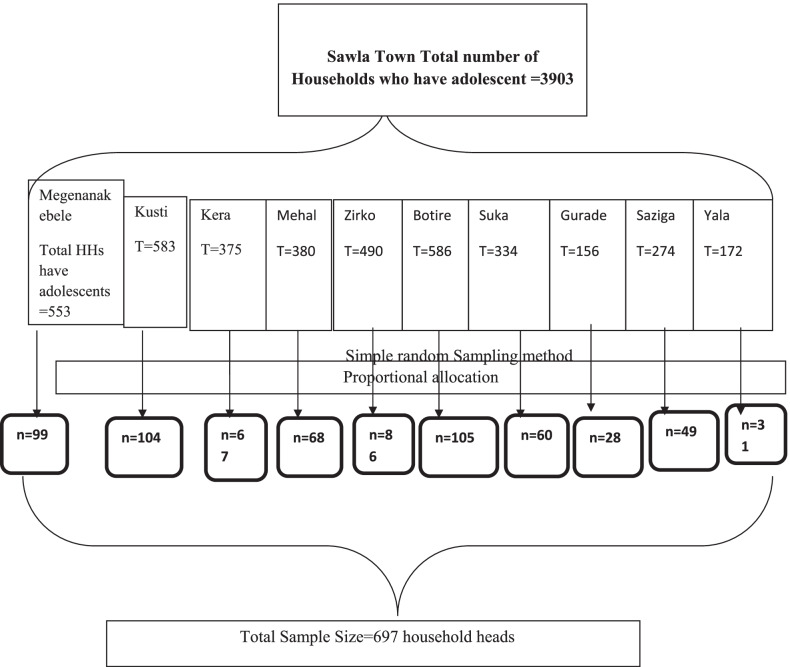
Schematic representation of the sampling procedure of parent–adolescent discussion and associated factors on SRH issues among parents in Sawla town, Southern Ethiopia, 2020

A simple random sampling method was employed to select the households. The number of households included in each kebele, the smallest administrative unit, is determined in proportion to the total number of households in each kebele. A computer generated random numbers were used to randomly select households from the lists of households who have adolescents (Fig. 1).

### Data collection and measurement

Data was collected using pretested structured questionnaire, adapted from EDHS 2016 and other published literature [21, 22]. The questionnaire consists of questions about socio-demographic factors, knowledge about SRH issues which was assess using eight questions, attitude towards SRH issues assessed using seven likert scale questions and discussion between Parents and Adolescents concerning SRH issues. Knowledge about SRH issues was assess using eight yes or no type questions. Attitude towards SRH issues was assessed using seven Likert scale questions which ranges from strongly disagree to strongly agree and finally dichotomized as positive and negative attitude.

Face to face interviews were conducted by six experienced health professionals, supervised by public health officer from the town health office. Data collectors and supervisor were trained for two days through practical exercises.

### Operational definitions

#### Parent–adolescent discussion on SRH issues

Parents who discussed at least two SRH issues (physical and psychological changes during puberty, sexual intercourse, condom, STI/HIV/AIDS, unwanted pregnancy, contraception) with adolescents in the last 6 months [21, 23].

#### Knowledgeable on SRH

Those parents who scored above the mean of the sexual and reproductive health knowledge related questions [21, 24].

#### Positive attitude about SRH issues

Those parents who scored above the mean of the SRH knowledge related questions [21].

### Data quality and management

Questionnaire was prepared in English language, translated to local language (Goffatho) and back translated to English by an expert for consistency. Questionnaire was pre-tested on 5% of the calculated sample size among parents from Galma Town which is not included in the actual study to ensure clarity of the questionnaire and then the necessary modifications and correction was made to standardize and ensure its validity. Adjustments in the sequence and wording were made based on the results of the pre-test. Questionnaire was checked for completeness on a daily basis. Incorrectly filled or missed questionnaire were sent back to respective data collector for correction. Principal investigator would recheck the completed questionnaires.

### Data processing and analysis

The collected data was coded and entered in to Epi-Data Version 3.1 [25] and analyzed using SPSS Version 21 software [26]. Descriptive analysis such as proportions, measure of central tendency and dispersion were computed for relevant variables. Model fitness was tested using Hosmer and Lemeshow test (> 0.05).All variables with p-value less than 0.25 in bivariate analysis were entered into multiple logistic regression model to test for association with the dependent variable at 95% confidence level (CI). P-value less than 0.05 and 95% CI for adjusted odds ratios (AOR) were used to declare the significance of the associations with the outcome variable.

## Results

### Socio-demographic characteristics of respondents

The response rate was 94% (653 of 697 parents participated). The mean age of respondents was 42 years (standard deviation (SD) 6.60). Nearly 80% of the respondents were Christian (521) and ethnically Gofa (518) Nearly a quarter (23.4%, 153) of the respondents had no formal education while 34.8% (227) were civil servants. One hundred forty-five (22.2%) had monthly income of 1000–2000 Ethiopian Birr. The majority of respondents were married (581, 89%) and a parent; only 1.7% (11) were guardians (Table1).

**Table 1:** Socio demographic characteristics of participants of parent–adolescent discussion about SRH issues study in Sawla town, Southern Ethiopia 2020 (n = 653)

Variable	Category	Frequency	%
Age	< 35	62	9.5
	35–45	405	62.0
	> 45	186	28.5
Sex	Male	322	49.3
	Female	331	50.7
Religion	Christian	521	79.8
	Muslim	132	20.2
Ethnic group	Gofa	518	79.3
	Others^a^	135	20.7
Educational level	No formal education	153	23.4
	Primary School	99	15.2
	Secondary school	129	19.8
	Diploma and above	272	41.7
Occupation	Daily labors	119	18.2
	Civil servants	227	34.8
	Private sector employee	108	16.5
	Merchant	199	30.5
Monthly income	< 1000	25	3.8
	1000–2000	145	22.2
	> 2000	483	74.0
Marital status	Single	10	1.5
	Married	581	89.0
	Divorced	44	6.7
	Widowed	18	2.8
Relation with adolescent	Father	320	49.0
	Mother	322	49.3
	Guardian	11	1.7

^a^Gamo, Wolayta, Oyda

### Knowledge towards SRH issues

Concerning participants’ knowledge of SRH issues about 72% (473) were considered to have good knowledge. Of the total 653 respondents, almost all knew about at least one contraceptive method, 641 (98.2%). When asked separately, majority knew about injectable (96%), condom (95.4%), pills (91%) and natural safe period (standard days method) (70.9%). Almost all (98%) participants knew at least one STI including HIV. Specifically, majority knew about HIV (98.3%) and Syphilis (89.9%) followed by Cancroid (66.5%). Almost all respondents knew at least one physical and behavioral change during puberty 649(99.4%). They mentioned breast development, onset of menstruation in females, change in voice in both sexes, refusal of parent command, feeling angry for minor reasons and interest towards opposite sex 99.1%, 98.3%, 96.6%, 77.3%, 73.2% and 71.7%, respectively (Table 2).

**Table 2:** Knowledge of parents on parent–adolescent discussion about SRH issues in Sawla town, Southern Ethiopia 2020 (n = 653)

Variables	Category	Frequency	%
Knowledge about SRH issues	Good	473	72.4
	Poor	180	27.6
Ever heard of SRH	Yes	629	96.3
	No	24	3.7
Knew at least one contraceptive method	Yes	641	98.2
	No	12	1.8
Type of contraceptive^a^	Pill	594	91.0
	Condom	623	95.4
	Injectable	627	96.0
	Implants	284	43.5
	IUCD	256	39.2
	Natural safe period	463	70.9
Knew at least one STI including HIV	Yes	640	98.0
	No	13	2.0
Type of STI/HIV^a^	Chancroid	434	66.5
	Syphilis	587	89.9
	Gonorrhea	310	47.5
	Lympho granuloma venerum	154	23.6
	HIV/AIDS	642	98.3
	Herpes simplex	86	13.2
Knew at least physical and behavioral change during puberty	Yes	649	99.4
	No	4	0.6
Type of physical and behavioral changes during puberty^a^	Breast development in female	647	99.1
	On set of menstruation in females	642	98.3
	Change in voice in both sex	631	96.6
	Refuse parent command	505	77.3
	Interest towards opposite sex	468	71.7
	Feel angry for minor reasons	478	73.2

^a^Multiple choices was possible

### Attitude about SRH issues

Overall, 582 (89.1%) participants had positive attitude towards SRH issues. Majority of respondents 447(68.50%) strongly disagree accepting premarital sex, while 5 (0.80%) had no problem with having premarital sex. About the same percent of parents (55%) agreed that both girls and boys must remain a virgin until they are married. Of the total 653 respondents, 400 (61.3%) believed that parent to adolescent discussion on SRH issues can delay first sexual intercourse. Four hundred sixty-four (71.10%) of parents believed that if unmarried couples want to have sexual intercourse before marriage they must use condom. Of all, 442 (67.70%) believed that HIV and other STIs can be prevented by using condom during sexual intercourse. About half of the participants (332) reported that their culture forbids parent to adolescent discussion about SRH issues.

### Discussion on SRH issues

**Fig. 2:**
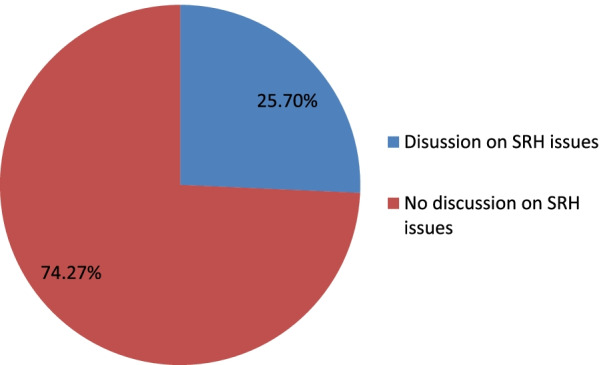
Distribution of parent–adolescent discussion on SRH issues in Sawla town, Southern Ethiopia 2020 (n = 653)

The study showed that 485 (74.27%) of the total participants did not have any discussion on SRH issues (Fig. 2).

Topics discussed between parents and adolescents about SRH issues were puberty (54, 8.3%), sexual intercourse (156, 23.9%), contraception (46, 7.0%), condom use (24, 3.7%), STI (138, 21.1%) and unintended pregnancy (120, 18.4%) (Table 3).

**Table 3:** SRH topics discussed between parents and adolescents in Sawla town, Southern Ethiopia 2020 (n = 653)

Variable	Category	Frequency	%
Puberty discussion^a^	Yes	54	8.3
	No	599	91.7
Sexual intercourse^a^	Yes	156	23.9
	No	497	76.1
Contraception^a^	Yes	46	7.0
	No	607	93.0
Condom^a^	Yes	24	3.7
	No	629	96.3
STI^a^	Yes	138	21.1
	No	515	8.9
Unintended pregnancy^a^	Yes	120	18.4
	No	533	81.6

^a^Multiple choices was possible

### Factors associated with parent to adolescent discussion on SRH issues

On bivariate logistic regression religion, educational status, occupation, monthly income, marital status, knowledge and attitude about SRH issues were found to be significantly associated with discussion on SRH issues (Table 4).

**Table 4:** Bivariate analysis of parent to adolescent discussion on SRH issues in Sawla town, Southern Ethiopia, 2020

Variables	Category	Parent–adolescent SRH discussion		Crude odds ratio (COR) (95%CI)	p-value
		Yes (%)	No (%)		
Religion	Christian	137 (26.3)	384 (73.7)	1	
	Muslim	31 (23.5)	101 (76.5)	0.88 (0.56, 1.38)	0.059
Marital status	Married	152 (26.2)	429 (73.8)	1	
	Divorced	6 (13.6)	38 (86.4)	0.39 (0.19, 0.78)	0.001
	Single	2 (20.0)	8 (80.0)	0.49 (0.24, 0.99)	0.002
	Widowed	8 (44.4)	10 (55.6)	1 (0.38, 1.62)	
Educational level	No formal education	7 (4.6)	146 (95.4)	1	
	Primary school	12 (12.1)	87 (87.9)	3.26 (1.24, 8.53)	0.002
	Secondary school	51 (39.5)	78 (60.5)	11.373 (5.00, 25.86)	< 0.001
	Diploma and above	98 (36.0)	174 (64.0)	29 (17.25, 52.96)	0.043
Occupation	Daily laborer	5 (4.2)	114 (95.8)	1	
	Civil servant	76 (33.5)	151 (66.5)	22.30 (8.50, 58.40)	0.001
	Private sector employee	56 (51.9)	52 (48.1)	5.16 (2.00, 13.33)	< 0.001
	Merchant	31 (15.6)	168 (84.4)	3.74 (1.40, 9.97)	0.002
Monthly income	< 1000	6 (24.0)	19 (76.0)	1	
	1000–2000	44 (30.3)	101 (69.7)	2.07 (1.16, 3.69)	0.023
	> 2000	118 (24.4)	365 (75.6)	7.35 (4.29, 12.62)	0.003
Knowledge about SRH	Poor	20 (11.1)	160 (88.9)	1	
	Good	148 (31.3)	325 (68.7)	3.64 (2.20, 6.03)	0.002
Attitude about SRH	Negative	6 (8.5)	65 (91.5)	1	
	Positive	162 (27.8)	420 (72.2)	4.18 (1.78, 9.83)	0.015

Multiple logistic regression model revealed that parents who have attended primary education were three times more likely to discuss SRH issues compared to those with no formal education (AOR 3, 95% CI: 1.10, 7.70). Parents who have attended secondary education were nine times more likely to discuss SRH issues compared to those with no formal education (AOR 9, 95% CI: 3.87, 20.64). The odds of discussion on SRH issues among adolescents and their parents was 22.3 times more likely among parents who attended higher education (diploma and above) (AOR 22.33, 95% CI: 9.49, 32.56).

Parents who reported a monthly income of above 2000 Ethiopian Birr were 2.4 times more likely to discuss SRH issues with their adolescents (AOR 2.40, 95% CI:1.30, 4.55) compared to parents with a monthly income less than 1000 Birr.

Parents who had good SRH knowledge were two-fold more likely to discuss SRH issues with their adolescents (AOR 2, 95% CI: 1.14, 3.50) than their counterparts. The odds of discussing SRH issues with their adolescents were 4 times higher among parents who had positive attitude than their counterparts (AOR 4, 95% CI: 1.57, 10) (Table 5).

**Table 5:** Multi-variable analysis of factors related with parent to adolescent discussion on SRH issues in Sawla town, Southern Ethiopia, 2020

Variables	Category	Parent–adolescent SRH discussion		COR (95%CI)	AOR (95%CI)	p-value
		Yes (%)	No (%)			
Educational level	No formal education	7 (4.6)	146 (95.4)	1	1	
	Primary school	12 (12.1)	87 (87.9)	3.26 (1.24, 8.53)	3 (1.10, 7.70)	0.001
	Secondary school	51 (39.5)	78 (60.5)	11.373 (5, 25.86)	9 (3.87, 20.64)	0.003
	Diploma and above	98 (36.0)	174 (64.0)	29 (17.25, 52.96)	22.33 (9.49, 32.56)	0.002
Monthly income	< 1000	6 (24.0)	19 (76.0)	1	1	
	1000–2000	44 (30.3)	101 (69.7)	2.07 (1.16, 3.69)	1.3 (0.70, 2.50)	
	> 2000	118 (24.4)	365 (75.6)	7.35 (4.29, 12.62)	2.4 (1.30, 4.55)	0.001
Knowledge about SRH	Poor	20 (11.1)	160 (88.9)	1	1	
	Good	148 (31.3)	325 (68.7)	3.64 (2.20, 6.03)	2 (1.14, 3.50)	≤ 0.001
Attitude about SRH	Negative	6 (8.5)	65 (91.5)	1	1	
	Positive	162 (27.8)	420 (72.2)	4.18 (1.78, 9.83)	4 (1.57, 10)	0.001

## Discussion

This study was conducted to assess parent to adolescent discussion about SRH issues from parents’ perspective including knowledge, reasons and associated factors.

In this study 25.7% (95% CI: 22.48%, 29.19%) parents reported that they had discussion on two or more topics in the last 6 months prior to the study about SRH issues with their adolescent children. This finding is similar to findings from other different regions of Ethiopia like Awabel Woreda in East Gojjam/Western Ethiopia (25.3%), Harar (28.7%) and Benishangul Gumuuz (28.9%). Similar findings were reported from China [27] which showed that the discussion rarely occurs despite accepting its importance. The results of this study are relatively higher than studies from Zeway, Ethiopia (20%) [28] and Lesotho (20%) [29]. This difference could have been due to the time gap as improvement in accessing SRH information may have occurred. This result is also lower than the findings in Mekele (57.6%) and Alamata (68.2%). The difference might be due to difference in measurement tools as study in Mekele considered discussion on one topic enough as compared to this study where at least two topics were used as cutoff. Similarly, different results from the study in Alamata may be due to the fact that, they considered discussions between sisters, brothers and health personals as parental discussion [13, 30]. When compared to results from studies in other continents the results were much lower. For example Mexico reported 83.1% parents have spoken to their children about SRH issues [11]. A study from New York, Alabama reported about the proportion to be 70.6% [9]. This might be due to difference in educational level of parents, knowledge, skill of communication and accessing sexual and reproductive health information which is better in Mexico and New York, Alabama compared to Ethiopia.

In this study discussion about puberty was 8.3%, which is similar to previous study done in Ziway (10.6%) [28]. However, this result was much lower than in the result reported from Yirgalem, southern Ethiopia (39.7%). This difference may be a result of difference in measuring tool as they considered one topic enough to constitute parental discussion. In addition time period was not restricted, which may have resulted in increased proportion [31]. The discussion about sexual intercourse (23.9%) was similar to study done in Debremarkos (27.6%) but much lower than the studies done in Yirgalem (33.5%), Bullen woreda of Benishangul Gumuuz (42.2%) and Harar (50.02%). This may be due to different in measurement criteria. In Bullen, discussion with teachers, friends, sisters and brothers was considered as being part of having parental discussion [21, 23, 31, 32].

Only 7% participants in this study reported discussion on contraception. This is lower than reported by other studies done in different parts of Ethiopia, including in Debremarkos (25.9%), Harar (26.39%), Yirgalem (36.1%) and Bullen woreda of Benishangul Gumuuz (41.3%). The difference may similar to as reported above, being difference in measurement criteria [21, 23, 31, 32]. Only 3.7% of the participants from this study discussed condom use and the result is far lower than other studies done in Ethiopia [21, 31, 32].

One fifth (21.1%) participants of this study discussed about STI and HIV/AIDS. This is lower than the study in Bullen woreda of Benishangul Gumuuz, Ethiopia (78.6%). This may be due to difference in study population and that they considered discussions between teachers, friends, sisters and brothers as parental discussion [32]. Eighteen percent of the respondents discussed about unintended pregnancy which is similar to the study from Ziway (20.7%) but much lower than other studies in Debremarkos (28.5%), Yirgalem (36.1%), Harar (42.59%) and in Bullen Benishangul Gumuuz (54.1%) This may be due to difference in measurement tools and study population [21, 23, 28, 31, 32].

Parents also indicated various reasons why they do not discuss sexual and reproductive health issues with their adolescents. Among the major reasons, cultural norms (54.2%) was mentioned which is in line with the study done in Ziway (54%) but greater than other studies done in Yirgalem, Harar and Bullen woreda of Benishangul Gumuuz [21, 23, 28, 31, 32].This shows existence of extensive cultural taboos throughout the country. Fear of discussion (41.5%) was also mentioned as one of the major reasons this is in line with many other researches [18, 23, 31, 33]. This may be due to the taboo nature of discussion about SRH issues in many Ethiopian settings.

Thirty-six percent of the respondents do not discuss due to lack of communication skills. This result is consistent with many studies done in different parts of Ethiopia and from abroad [18, 23, 31, 33, 34] indicating a gap in communication skills among parents. Belief that discussion would initiate sex (34%) was also mentioned as a reason, which is similar to the study done in Debremarkos (33.1%) and Harar (33.8%) where parents perceive that discussing sexual matters with their adolescents might encourage the children to engage in premarital sex [21, 23]. Some of the participants also mentioned lack of knowledge (20.8%) as a reason for no discussion. This result is similar to the study done in Wollega and Benishangul Gumuuz [32, 33]. This may be due to parents’ perspective that the adolescents know more than them.

Majority of the participants had good knowledge about SRH issues (72.4%) (95% CI: 68.91%, 75.76%). This is greater than the studies done in different parts of Ethiopia like, Harar (67.38%) and Debremarkos (57.3%). This difference may be due to time gap between the studies which may have resulted in improvements in available resources [21, 23].

The result of multiple logistic regression models revealed that parents who had no formal education were less likely to discuss about SRH issues when compared to those parents who had primary education (AOR 3, 95% CI: 1.1, 7.7), secondary education (AOR 9, 95% CI: 3.87, 20.64) and diploma and above (AOR 22.33, 95% CI: 9.49, 32.56).This result is consistent with different previous studies [23, 33–36]. This may be due to educated parents have better information about SRH issues and communication skills, so that they are more likely to discuss than those with no formal education. Knowledge leads to the development of desirable attitude and builds confidence in parents to shoulder the matter.

Parents who reported a monthly income of above 2000 Ethiopian Birr were 2.4 times more likely to discuss SRH issues with their adolescents (AOR 2.4, 95% CI:1.3, 4.55) compared to parents with a monthly income of less than 1000 Birr. This is similar to the study done in Harar [23]. Parents who had good SRH knowledge had a two fold increase in the odds of discussing SRH issues with their adolescents (AOR 2, 95% CI: 1.14, 3.5) than their counterparts. This result was consistent with previous findings [23, 33, 34, 36]. This may be due to respondents who had good knowledge were eager to discuss and their knowledge may initiate discussion and they may have interest to share their knowledge. The odds of discussion on SRH issues were 4 times higher among parents who had positive attitude than their counterparts (AOR 4, 95% CI: 1.57, 10).This result was also consistent with previous findings [23, 33, 34, 36]. This may be due to their perceived importance of discussion for their adolescents.

### Strengths and limitations of the study

This study is community based and conducted from parents’ perspective unlike many researches done from adolescent perspective. However, the study was based on self-reporting and responses might be affected by social desirability bias because of the sensitive nature and cultural barriers for reporting. Since the study design was cross sectional, cause and effect relationship could not be established. The presumption that discussion is positive, when in reality, some discussions can be quite negative and harmful is limitation of the study. Another limitation is that only adults were interviewed. It would have been interesting to get the adolescents' perspective and see if it aligned with the parents.

### Research policies and implications

As a primary socializing agent, and as live model, parents are very important in shaping the current and the future lives of their adolescents. Indeed, this study provides important programmatic implications for amending policies and programs which address cultural and social factors that influence parental discussion about SRH issues.

## Conclusions and recommendations

Different evidences suggest that parent to adolescent discussion on reproductive health issues is the basis for safe and healthy transition to adulthood. However, in this study even if parents had good knowledge and positive attitude, the proportion of discussion about sexual and reproductive health is low. This is bounded by different cultural taboos, fear of discussion and lacks of communication skill and belief that talking about sex will initiates sex. Parents who attended primary education had better chance of discussing on SRH issues with adolescents. Those with higher monthly income had discussed on SRH with adolescents. Similarly, those with good knowledge and positive attitude also had discussed about SRH with their adolescents than their counterparts. We recommend provision of continuous capacity building on SRH issues for parents to increase the level of parental knowledge and attitude about SRH issues and sustainable advocacy should be provided about the importance of parent to adolescent discussion. Moreover, it is important to support and scale up the adult learning program to decrease the level of illiteracy. In the next AYRH Strategy update education for parents on SRH issues and skills development for empowering them to have SRH discussions with their adolescents. Another recommendation would be to engage health providers with the tools and job aids to help them discuss the importance of parent–adolescent communication on SRH issues, and including activities around this topic in donor-funded AYRH projects in Ethiopia. Finally, research is needed to fully understand the issues relevant to social meanings and beliefs that were major reason for low discussion between parent and adolescent.

## Data Availability

The datasets during the current study are available from the corresponding author on reasonable request.

## Abbreviations

AIDS: Acquired immune deficiency syndrome
AOR: Adjusted odds ratio
CI: Confidence interval
COR: Crude odds ratio
EDHS: Ethiopian Demographic and Health Survey
HIV: Human immune virus
HH: House holds
IUCD: Intra uterine contraceptive device
KMs: Kilometers
SD: Standard deviation
RH: Reproductive health
SPSS: Statistical Package for Social Science
SRH: Sexual and Reproductive Health
STD: Sexually transmitted disease
STI: Sexually transmitted infection
UNFPA: United Nation Population Fund
WHO: World Health Organization
